# Adoption of improved management practices of livestock: Case of small-scale farmers in rural Bangladesh

**DOI:** 10.1016/j.heliyon.2023.e18667

**Published:** 2023-07-25

**Authors:** Md Sadique Rahman, Md Hayder Khan Sujan, Md Sherf-Ul-Alam, Monira Sultana, Mst Shopna Akter

**Affiliations:** a Department of Management and Finance, Sher-e-Bangla Agricultural University, Sher-e-Bangla Nagar, Dhaka, Bangladesh; b Department of Development and Poverty Studies, Sher-e-Bangla Agricultural University, Sher-e-Bangla Nagar, Dhaka, Bangladesh; c Department of Agricultural Economics and Policy, Sylhet Agricultural University, Sylhet, Bangladesh; d Department of Agricultural Economics, Sher-e-Bangla Agricultural University, Dhaka, Bangladesh

**Keywords:** Adoption, Artificial insemination, Concentrate feed, Livestock farming, Multivariate probit

## Abstract

This study examines the drivers of improved livestock management practices (ILMP) adoption in rural Bangladesh using data from the International Food Policy Research Institute's (IFPRI) Bangladesh Integrated Household Survey (BIHS). This study investigated four improved management practices: artificial insemination, concentrate feed, vaccination, and deworming. The binary logit and multivariate probit (MVP) models were used to analyse the data. According to the findings, approximately 65% of farmers practiced deworming, but only about 20% of farmers vaccinated their livestock. Logit regression analysis suggested that rural households with mobile phones and televisions were 5.2% and 3.8% more likely to adopt. Furthermore, compared to their peers, farmers who have maintained contact with livestock extension agents are 11% more likely to adopt. The MVP model indicated that likelihood of using concentrate feed increases with income, while artificial insemination is more prevalent among farmers who raise livestock for sale. Policy implication included the need for more extension agent-delivered awareness development programmes to educate livestock farmers on the benefits of ILMP. Scale-appropriate management practices can also play an important role. Farmers should be guaranteed of the availability of livestock feed and veterinary services at reasonable prices to promote adoption.

## 1 Introduction

Livestock is a crucial part of many farming systems and provides smallholder farmers with animal protein, farm power services, income, and employment opportunities [1]. Livestock sector provides employment for over 6 million people in Bangladesh [2]. Although the contribution of overall agricultural production to total Gross Domestic Product (GDP) growth rate has decreased from 50.9% to 13.2% between 1973 and 2020, the contribution of the livestock sector to GDP growth rate has remained consistent, ranging between 2.1 and 3.6% [3]. The livestock sector can considerably contribute to national income. Currently, Bangladesh's per capita meat consumption is 36.93 g per day, which is lower than the World Health Organization's (WHO) recommended intake of 120 g per day and capita [4]. Similarly, per capita milk consumption is 175.63 ml per day, compared to the recommended daily consumption of 250 ml per day [4]. Demand for livestock products, however, has grown in recent years as a result of rising incomes, changing diets, and increased urbanization. Therefore, there is a renewed need to increase livestock production in order to fulfil the rising demand for livestock-based products. Adopting ILMP can significantly facilitate the increase of livestock production. However, most of the small-holder farmers in rural Bangladesh raise cattle using traditional methods [5].

Farmers in traditional farming systems rely on local livestock breeds and local communally managed pastures, which results in low productivity, and high mortality [6]. However, farmers have recently begun adopting ILMP in Bangladesh. For instance, a study found that approximately 24% of farmers in Bangladesh utilized the vaccine for foot and mouth disease. Adopting ILMP can help to reduce disease infestation and mortality rates while increasing productivity [7]. However, the decision to adopt any farming-related technology is complicated [8]. A variety of factors, including socio-demographic features, farm characteristics, economic conditions, and institutional factors, may influence decision making [[9], [10], [11], [12]]. As a result, identifying the factors' influencing adoption is critical for the livestock sector's development.

Previous research revealed that communication between farmers and technicians is essential for the adoption of livestock management practices in Brazil [9,10]. Adoption of ILMP is also influenced by education, cattle number, and information source [11,12]. A few studies conducted in Africa found that off-farm income, herd size, and education played important roles in adoption decision [[13], [14], [15]]. It is evident that the majority of previous studies were conducted in the Sub-Saharan African context. In addition, several studies analyzed data using a small sample size [9,10,12]. Several other studies ignored potential synergies among ILMP and argued that they are mutually exclusive [16,17]. Using a robust econometric model, only a limited number of studies have identified the factors influencing the adoption of ILMP in the South Asian context, particularly in Bangladesh.

Our study contributes to the current literature in two ways. First, using a nationally representative sample, this study identifies the drivers of adoption of ILMP, which may then be applied to other nations with similar contexts. Second, to identify the determinants influencing adoption, this study used a multivariate probit (MVP) model, which acknowledges the potential synergy among ILMP. The identification of adoption-influencing factors aims to fill policymakers' knowledge gaps, thereby enhancing the ongoing success of livestock development programmes.

## 2 Methodology

### 2.1 Data sources

The study used BIHS data to achieve its objective. IFPRI conducted this household survey in rural Bangladesh during 2018–2019 [18]. The BIHS used stratified sampling technique to select the households. Eight strata were created and 325 villages were selected from these 08 strata based on household percentage. Twenty households were selected from each village for face-to-face interview. However, a few households refused to participate in the final survey and could not be located. Finally, 5605 households from rural Bangladesh were surveyed. In this study households that did not raise livestock were excluded from the analysis, which led to a sample size of 2317 households.

In Bangladesh, ILMP refers to diverse management practices including housing system, feeding strategies, artificial insemination, concentrate feed, pasture management, vaccination, and deworming. In this study, however, we evaluated four ILMP, including artificial insemination, concentrate feed, vaccination, and deworming. The selection of these four practices was based on the fact that BIHS data contain specific information about them. The BIHS lacked detailed data on other ILMP. Artificial insemination is a prevalent type of reproductive technology, and farmers utilize it on their farms because of its efficiency [19,20]. Commercial artificial insemination services have been provided in Bangladesh by the government and non-government organizations. Concentrate feeding is regarded as an efficient technique that includes high energy value items such as fat and cereal grains, as well as high-protein oil meals or cakes. Rice polish, wheat bran, legume bran, salt, molasses, and oil cakes make up the concentrate feed in rural Bangladesh. Deworming reduces the prevalence of parasites in the gastrointestinal tract [21,22]. Livestock farmers are facing issues due to productivity losses caused by livestock diseases. As a result, livestock vaccination is regarded as an improved management approach that has the potential to minimize the burden of infectious diseases. Farmers in Bangladesh administer both deworming and vaccination based on the prescriptions of veterinarians.

### 2.2 Model specification

The study employed binary logit and multivariate probit (MVP) model to assess the relationship between adoption of ILMP and farmers’ characteristics. Random utility framework was followed to assess the relationship. Under this framework, a farmer will adopt an ILMP if the utility gain from adoption is higher than non-adoption [23]. For logit model, the dependent variable was binary and a value of 1 was given if a farmer adopted any of the ILMP, otherwise, 0. Following previous studies [24,25], the probability of adoption can be modelled as shown in Eq. (1):

(1) $$\mathrm{Pr}\left(adoption=1\right)=\frac{\exp\left(\beta Z_{i}\right)}{1+\exp\left(\beta Z_{i}\right)}$$

Where, Z_i_ represents explanatory variables and ***β*** represents parameters to be estimated.

The probability of non-adoption can be modelled as shown in Eq. (2):

(2) $$\mathrm{Pr}\left(non-adoption=0\right)=\frac{1}{1+\exp\left(\beta Z_{i}\right)}$$

Thus, the ratio between two probabilities can be obtained as shown in Eq. (3):

(3) $$\frac{\mathrm{Pr}\left(adoption=1\right)}{\mathrm{Pr}\left(non-adoption=0\right)}=\exp\left(\beta Z_{i}\right)$$

The empirical logit model is given in Eq. (4):

(4) $$Y_{i}=\beta_{0}+\sum_{i=1}^{15}\beta_{i}Z_{i}+u_{i}$$

Where, $Y_{i}$ represent adoption status (adoption = 1, otherwise 0), $\beta_{0}$ = Constant, $\beta_{i}$ is parameters to be estimated, Z_i_ represents explanatory variables, and $u_{i}$ = error term.

The binary logit model ignores interdependent and simultaneous adoption decisions, which may produce bias results [26]. A binary model also assumes independence of error terms [27]. Adoption of one ILMP may influence multiple others. Thus, the MVP model was used to identify drivers of multiple ILMP adoption. The MVP model simultaneously estimated the factors influencing adoption of the four ILMP by allowing their error terms to correlate. In the MVP model, the error terms jointly follow a multivariate normal distribution with zero conditional mean and variance normalized to unity. The resulting covariance matrix (Σ) is given in Eq. (5):

(5) $$\Sigma =\left[\begin{array}{cccc} 1 & \rho_{12} & \rho_{13} & \rho_{14}\\ \rho_{21} & 1 & \rho_{23} & \rho_{24}\\ \rho_{31} & \rho_{32} & 1 & \rho_{34}\\ \rho_{41} & \rho_{42} & \rho_{43} & 1 \end{array}\right]$$

Where, ρ represent the pairwise correlation coefficient of the error terms corresponding to any two improved management practices. The nonzero value of ρ justifies the use of MVP model instead of a binary logit or probit for each individual improved management practice.

### 2.3 The variables

Based on prior researches [9,11,13,25,28,29], this study considered 15 potential explanatory variables in determining the adoption of ILMP. The description of the explanatory variables is given in Table 1.

**Table 1:** Description of explanatory variables.

Variable	Description
Age (Years)	Age of the household's head in years.
Education (Years)	Years of education attained by the household's head.
Gender (dummy)	1 if household head is male, otherwise 0.
Spouse education (Years)	Years of education of household head's spouse.
Working member (Number)	Number of working members in the household.
Mobile phone (dummy)	1 if household owns a mobile phone, otherwise 0.
Television (dummy)	1 if household owns a television, otherwise 0.
Annual income (USD)	Yearly household income in USD.
Access to electricity (dummy)	1 if household has access to electricity, otherwise 0.
Crop farm size (ha)	Total land under crop farming in ha.
Extension contacts (dummy)	1 if livestock extension agent visited the household for livestock farming advice over the last 12 months, otherwise 0.
No. Of livestock (Number)	Total number of cattle owned by a household.
Distance from market (km)	The distance between the respondent's house and local market.
Livestock raised for marketing only (dummy)	1 if purpose of livestock farming is only selling, otherwise 0.
Livestock raised for marketing and consumption (dummy)	1 if purpose of livestock farming is selling and consumption, otherwise 0.

## 3 Results

### 3.1 Adoption status

**Fig. 1:**
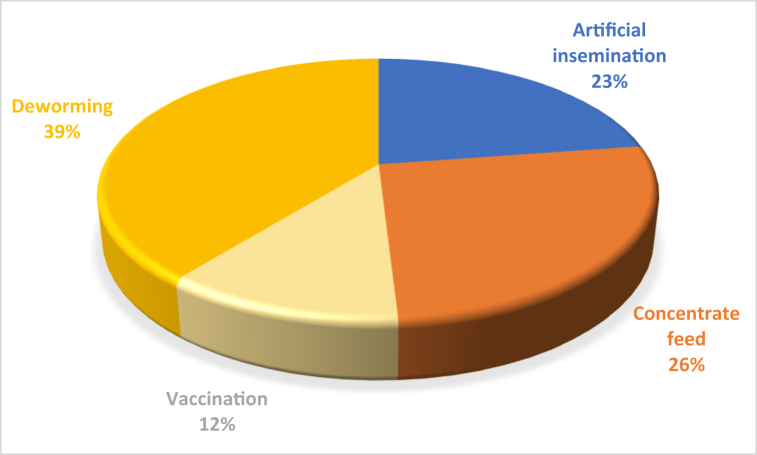
Percentage of farmers adopting different ILMP.

**Fig. 2:**
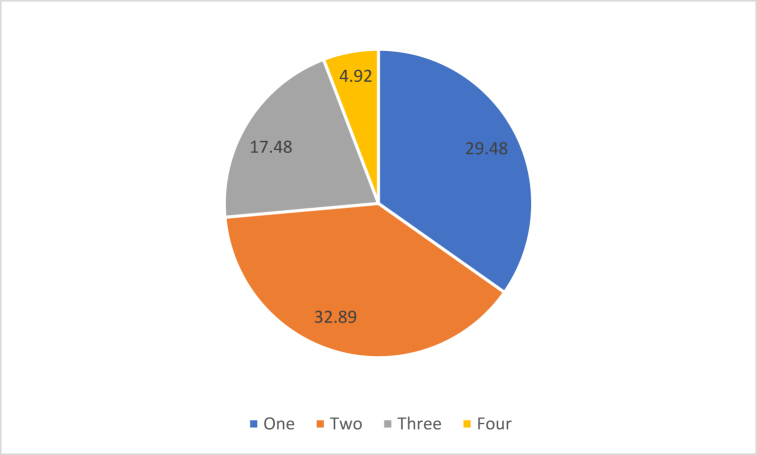
Percentage of farmers adopting number of ILMP.

Fig. 1 represents the adoption status of the selected ILMP. It is revealed from the table that deworming was the mostly adopted (65.65%) ILMP followed by concentrate feed (44.24%), artificial insemination (37.89%) and vaccination (19.59%). Fig. 2 indicated that most of the farmers (62.37%) adopted 1 or 2 practices. Only a few farmers adopted all the four practices.

### 3.2 Descriptive statistics of the explanatory variables

Table 2 represent the descriptive statistics of the variables used in the models. The mean age of farmers was found to be significantly higher for adopters than non-adopters. Most of the households were headed by male. Furthermore, adopters had a higher average number of working members in their households (4.61) than non-adopters (4.31). About 90% of adopters owned mobile phones and 41% had televisions in their households. In contrast, 86% and 29% of non-adopters owned a mobile phone and television, respectively. The findings also revealed that the annual income of adopters (USD 1845) was significantly higher than that of non-adopters (USD 1588). In terms of receiving extension services, there was a significant difference between adopters and non-adopters. About 16% of adopters received livestock extension services, compared to just 4% of the non-adopters. The average herd size of adopters was 2.74, compared to 1.82 for non-adopters. Seventy-eight percent of adopters, compared to about 65% of non-adopters, raised livestock for both sale and consumption.

**Table 2:** Descriptive statistics of the variables.

Variables	Adopters (n = 1964)		Non-adopters (n = 353)		Mean difference
	Mean	Standard deviation	Mean	Standard deviation	
Age (Years)	47.87	12.76	45.95	13.17	1.92***
Education (Years)	3.61	5.34	3.11	5.03	0.50
Gender (dummy)	0.91	0.29	0.87	0.33	0.04
Spouse education (Years)	3.70	9.56	3.37	10.93	0.33
Working member (Number)	4.61	1.83	4.31	1.59	0.30***
Mobile phone (dummy)	0.90	0.30	0.86	0.34	0.04**
Television (dummy)	0.41	0.50	0.29	0.49	0.12***
Annual income (USD)	1846	1633	1588	1474	258***
Electricity (dummy)	0.86	0.35	0.79	0.41	0.07***
Crop farm size (ha.)	0.41	0.52	0.39	0.53	0.02
Extension contacts (dummy)	0.16	0.36	0.04	0.20	0.12***
No. Of livestock (Number)	2.74	2.40	1.82	1.82	0.92***
Livestock raised for marketing (dummy)	0.20	0.40	0.32	0.47	−0.12***
Livestock raised for marketing and consumption (dummy)	0.78	0.42	0.65	0.48	0.13***
Distance from market (Kms.)	9.92	9.98	10.52	25.12	−0.60

### 3.3 Factors affecting adoption

Table 3 represents the results of adoption analysis using the binary logit model. The significant value of Wald Chi square shows the validity of model specification. Age, number of working members, ownership of a mobile phone, ownership of a television, access to electricity, extension contact, and number of livestock were found to have a positive and significant influence on the adoption of ILMP, whereas crop farm size and distance from market had a negative influence. For example, a one-year increase in age, while all other factors remain constant, increased the likelihood of adoption by 0.1%. Similarly, having one more working person increased the likelihood of adoption by 0.7%. The likelihood of adoption is increased by 5.2% and 3.8%, respectively, in households with a mobile phone and a television, compared to those without. Similarly, farmers who maintain contact with extension agents had a 10.8% higher likelihood of adoption. Keeping other things constant, a 1-ha increase in crop farm size reduces the likelihood of ILMP adoption by 3% (Table 3).

**Table 3:** Drivers of adoption via binary logit model.

Variables	Coefficients	SE	Marginal effect
Age (Years)	0.011**	0.005	0.001
Education (Years)	0.019	0.016	0.002
Gender (dummy)	0.066	0.197	0.007
Spouse education (Years)	−0.002	0.009	0.000
Working member (Number)	0.066*	0.038	0.007
Mobile phone (dummy)	0.416**	0.190	0.052
Television (dummy)	0.345**	0.149	0.038
Annual income (USD)	0.000	0.000	0.000
Electricity (dummy)	0.327**	0.160	0.039
Crop farm size (ha.)	−0.273**	0.120	−0.030
Extension contacts (dummy)	1.418***	0.283	0.108
No. Of livestock (Number)	0.220***	0.042	0.024
Livestock raised for marketing (dummy)	0.015	0.405	0.002
Livestock raised for marketing and consumption (dummy)	0.497	0.398	0.060
Distance from market (Kms.)	−0.004**	0.002	0.000
Constant	−0.809	0.554	0.001
Log pseudolikelihood	−912		
Wald chi-square	111***		
Pseudo R^2^	0.08		
No. Of observations	2317		

Table 4 shows the pairwise correlation coefficients between the four ILMP. Vaccination and deworming correlated with artificial insemination. Vaccination also strongly influenced deworming. The MVP model found that age, education, gender of the household head, television ownership, annual income, extension contact, number of livestock, and farming purpose positively influenced the decision to use artificial insemination (Table 5). The ownership of a mobile phone, television, annual income, and number of livestock positively influenced adoption of concentrate feed, whereas crop farm size negatively influenced adoption. Vaccination adoption was favourably influenced by the number of working members in the household, the extension contact, and the number of livestock. Similarly, the number of working members in the household, extension contact, number of livestock, and purpose of livestock farming all had a positive influence on the adoption of deworming.

**Table 4:** Pairwise correlation coefficients between ILMP.

Adaptation strategies	Correlation coefficients	Standard error
Artificial insemination and concentrate feed	0.037	0.032
Artificial insemination and vaccination	0.185***	0.036
Artificial insemination and deworming	0.291***	0.032
Concentrate feed and vaccination	0.021	0.037
Concentrate feed and deworming	0.030	0.032
Vaccination and deworming	0.371***	0.035

**Table 5:** Drivers of adoption via multivariate probit model.

Variables	Improved livestock management practices							
	Artificial insemination		Concentrate feed		Vaccination		Deworming	
	Coefficients	SE	Coefficients	SE	Coefficients	SE	Coefficients	SE
Age (Years)	0.006***	0.002	0.004	0.002	−0.004	0.003	0.001	0.002
Education (Years)	0.019***	0.005	0.008	0.005	0.003	0.006	0.001	0.005
Gender (dummy)	0.176*	0.100	−0.008	0.096	−0.077	0.113	0.136	0.097
Spouse education (Years)	−0.005	0.003	0.004	0.003	−0.004	0.004	0.004	0.003
Working member (Number)	−0.039**	0.016	0.027*	0.016	−0.038**	0.018	0.050***	0.017
Mobile phone (dummy)	−0.010	0.093	0.414***	0.094	0.098	0.109	0.174*	0.091
Television (dummy)	0.141**	0.057	0.121**	0.056	0.024	0.065	0.065	0.059
Annual income (USD)	0.001***	0.000	0.002***	0.000	0.000	0.000	0.000	0.000
Electricity (dummy)	0.413***	0.084	0.058	0.078	0.096	0.092	0.087	0.079
Crop farm size (ha.)	−0.042	0.057	−0.100*	0.055	−0.030	0.064	−0.008	0.059
Extension contacts (dummy)	0.312***	0.078	0.025	0.077	0.813***	0.078	0.591***	0.088
No. Of livestock (Number)	0.064***	0.012	0.021*	0.012	0.073***	0.013	0.077***	0.013
Livestock raised for marketing (dummy)	0.712***	0.195	−0.161	0.190	0.156	0.259	0.838***	0.197
Livestock raised for marketing and consumption (dummy)	0.044	0.186	−0.055	0.184	0.335	0.251	0.932***	0.191
Distance from market (Kms.)	0.000	0.002	0.000	0.001	0.002	0.001	−0.001	0.002
Constant	−0.906***	0.258	−0.964***	0.253	−1.613***	0.318	−1.438***	0.261
Log likelihood	−5343							
Wald chi-square	546***							
Loglikelihood test	181***							
No. Of observations	2317							

## 4 Discussion

According to descriptive statistics, the average age of adopters was higher than that of non-adopters. Higher age also signifies more experience, which may have an influence on technology adoption decisions [28,29]. Descriptive statistics also revealed that more than 90% of adopting households are headed by a man. When compared to non-adopters, adopters have better access to electricity, mobile phones, and television. Households with these amenities typically have better access to information, which may increase adoption**.** Farmers' acquaintance with and understanding of new technologies are important factors in assessing them, and access to relevant information can be beneficial in this regard [29].

Descriptive analysis also shows that adopters had a slightly higher annual income than non-adopters. Previous studies suggested that higher income can help finance the purchase and adoption of ILMP [13,29]. Our research also revealed that the majority of livestock farmers are small-scale farmers. Because of the small size of the herd, their income may be minimal, preventing them from purchasing feed and other veterinary services from the market, and as a result, the adoption rate is low. In addition, the descriptive statistics indicated that adopters had better extension contacts than non-adopters. However, there is plenty of opportunity for improvement in the extension activities, as only a small percentage of farmers are currently able to utilize them.

The adoption analysis suggested that the livestock farmers may have been reluctant to fully adopt all of the ILMP. The majority of livestock farmers dewormed their cattle. Lower adoption rates for concentrate feeding and vaccination were observed. Concentrate feed can boost livestock productivity, but few Bangladeshi farmers use it due to the high cost [30]. We found that most farmers have few cattle. Small herds may deter farmers from using more expensive concentrate feeds. Lack of information, vaccine, and expert personnel may deter farmers from adopting these practices.

The logit model results showed that age had a positive influence on overall adoption decision. Several prior studies have revealed that younger farmers adopt technologies at a higher rate than older farmers [28,29]. However, in our analysis, we found a positive association between farmers' age and adoption. The MVP analysis also revealed that the respondents' age had a significant positive influence on their adoption of artificial insemination. Farmers' expertise with livestock rearing, which is thought to be strongly connected with their age, may influence adoption decisions. A few prior research also found that age had a positive influence on the adoption of agricultural-related technology [13,31].

The logit model also showed that number of working members in the household positively influenced adoption. When ILMP were considered separately, the number of working members negatively affected artificial insemination and vaccination but positively affected feeding and deworming. Artificial insemination requires highly skilled people and specialized equipment [11], so family members are excluded from the process. Concentrate feed and deworming required human labour. Households with a larger number of members can participate in these activities, perhaps increasing the adoption of improved practices. Alternatively, households with more working members often earn a larger income and can invest in purchasing feed from the market, which may increase adoption.

Both models suggested that owning a mobile phone and television, which were used as proxies for information source and communication, positively influenced adoption. Households with mobile phones are more likely to communicate with peers and extension workers, which may increase the likelihood of adoption [1,25,32]. The regular broadcasting of television shows about livestock farming and the advantages of adopting ILMP can also foster a positive attitude among farmers. Adoption of concentrate feed was negatively impacted by crop farm size, which is consistent with the findings of Quddus [5]. This finding may be the result of several factors, one of which is that farmers with larger farm sizes are less likely to adopt feeding management practices because they are too busy growing crops. As a result, motivating and training farmers can encourage them to spend more time in livestock farming, which can increase the likelihood adopting ILMP.

According to both models, extension contacts have a positive and significant influence on adoption. The advice of livestock extension workers may increase farmers' knowledge and awareness, motivating them to adopt improved management practices. Previous studies also suggested that bolstering cooperation between extension agents and farmers can increase livestock sector development awareness [29,33]. As a result, extension activities such as field days, group discussions, and demonstrations can assist boost the adoption of improved management practices by increasing farmers' knowledge and understanding of the benefits of improved management practices.

The MVP model found that higher incomes were associated with higher usage of artificial insemination and concentrate feeding. Previous study also suggested that higher wealth promotes the use of more expensive practices [34]. When the government does not provide the artificial insemination services that livestock farmers need, they often have to pay a lot more to private companies. Therefore, farmers with higher incomes can spend more money on concentrate feed and on expert assistance with artificial insemination, which may lead to higher adoption.

The results also showed that the probability of adopting ILMP increased with the size of the operation, suggesting that farmers with more livestock were more likely to adopt. This finding is consistent with other studies' findings [11,35]. The findings also revealed that farmers who raised livestock solely for commercial purposes adopted artificial insemination and deworming. These associations could be due to a commercial purpose or an awareness of commercial livestock farmers. Artificially inseminated and dewormed livestock normally sell for a higher price, which may persuade farmers to adopt them.

## 5 Conclusions

There is a renewed need to increase livestock production as people become more health conscious and as household incomes rise. Adopting ILMP is a great way to help increase livestock production. This study identifies the factors that influences livestock farmers to adopt ILMP. Annual income, use of modern information and communication technologies, extension contacts, and herd size influenced farmers’ decision to adopt ILMP. The study emphasized the importance of extension services like trainings and demonstrations in promoting widespread adoption. By providing the necessary capital, livestock extension agents and concerned authorities can play an important role in encouraging farmers to engage in livestock farming. Due to the fact that the herd size and purpose of farming played a role in adoption, the creation and transfer of scale-appropriate management practices can also play a significant role in increasing adoption. The availability of high-quality feed and affordable veterinary services can aid in promoting adoption.

Despite the useful information obtained, there are limitations to this study. This study focuses on the adoption of four ILMP. Other ILMPs, including housing system and pasture management, were disregarded due to a lack of data. Future research may investigate all ILMP to obtain a complete picture. Dis-adoption of ILMP has emerged as a policy concern in recent years. Therefore, future research should incorporate the concept of dis-adoption to provide a more complete representation of the farm level. Future research could also consider utilizing multi-period panel data to obtain a more accurate estimate of factors influencing adoption.

## Author contribution statement

Md. Sadique Rahman: conceived and designed the experiment, analyzed and interpreted the data, wrote the paper.

Md. Hayder Khan Sujan: conceived and designed the experiment, wrote the paper.

Md. Sherf-Ul-Alam: contributed reagents, materials, analysis tools or data, wrote the paper.

Monira Sultana: contributed reagents, materials, analysis tools or data, wrote the paper.

Mst. Shopna Akter: contributed reagents, materials, analysis tools or data, wrote the paper.

## Data availability statement

Data will be made available on request.

## Additional information

Supplementary content related to this article has been publish online at [URL].

## Declaration of competing interest

The authors declare that they have no known competing financial interests or personal relationships that could have appeared to influence the work reported in this paper.
